# Machine learning enhances prediction of plants as potential sources of antimalarials

**DOI:** 10.3389/fpls.2023.1173328

**Published:** 2023-05-25

**Authors:** Adam Richard-Bollans, Conal Aitken, Alexandre Antonelli, Cássia Bitencourt, David Goyder, Eve Lucas, Ian Ondo, Oscar A. Pérez-Escobar, Samuel Pironon, James E. Richardson, David Russell, Daniele Silvestro, Colin W. Wright, Melanie-Jayne R. Howes

**Affiliations:** ^1^ Royal Botanic Gardens, Kew, Richmond, United Kingdom; ^2^ EaStCHEM, School of Chemistry, University of St Andrews, St Andrews, United Kingdom; ^3^ Gothenburg Global Biodiversity Centre, Department of Biological and Environmental Sciences, University of Gothenburg, Gothenburg, Sweden; ^4^ Department of Biology, University of Oxford, Oxford, United Kingdom; ^5^ UN Environment Programme World Conservation Monitoring Centre (UNEP-WCMC), Cambridge, United Kingdom; ^6^ School of Biological, Earth and Environmental Sciences, University College Cork, Cork, Ireland; ^7^ Tropical Diversity Section, Royal Botanic Garden, Edinburgh, United Kingdom; ^8^ Departamento de Biología, Facultad de Ciencias Naturales, Universidad del Rosario, Bogotá, Colombia; ^9^ Environmental Research Institute, University College Cork, Cork, Ireland; ^10^ Department of Biology, University of Fribourg, Fribourg, Switzerland; ^11^ Swiss Institute of Bioinformatics, Fribourg, Switzerland; ^12^ School of Pharmacy and Medical Sciences, University of Bradford, Bradford, United Kingdom; ^13^ Institute of Pharmaceutical Science, King’s College London, Franklin-Wilkins Building, London, United Kingdom

**Keywords:** malaria, traditional and indigenous knowledge, machine learning, botany, ethnobotany, sampling bias, antiplasmodial activity, ethnopharmacology

## Abstract

Plants are a rich source of bioactive compounds and a number of plant-derived antiplasmodial compounds have been developed into pharmaceutical drugs for the prevention and treatment of malaria, a major public health challenge. However, identifying plants with antiplasmodial potential can be time-consuming and costly. One approach for selecting plants to investigate is based on ethnobotanical knowledge which, though having provided some major successes, is restricted to a relatively small group of plant species. Machine learning, incorporating ethnobotanical and plant trait data, provides a promising approach to improve the identification of antiplasmodial plants and accelerate the search for new plant-derived antiplasmodial compounds. In this paper we present a novel dataset on antiplasmodial activity for three flowering plant families – Apocynaceae, Loganiaceae and Rubiaceae (together comprising c. 21,100 species) – and demonstrate the ability of machine learning algorithms to predict the antiplasmodial potential of plant species. We evaluate the predictive capability of a variety of algorithms – Support Vector Machines, Logistic Regression, Gradient Boosted Trees and Bayesian Neural Networks – and compare these to two ethnobotanical selection approaches – based on usage as an antimalarial and general usage as a medicine. We evaluate the approaches using the given data and when the given samples are reweighted to correct for sampling biases. In both evaluation settings each of the machine learning models have a higher precision than the ethnobotanical approaches. In the bias-corrected scenario, the Support Vector classifier performs best – attaining a mean precision of 0.67 compared to the best performing ethnobotanical approach with a mean precision of 0.46. We also use the bias correction method and the Support Vector classifier to estimate the potential of plants to provide novel antiplasmodial compounds. We estimate that 7677 species in Apocynaceae, Loganiaceae and Rubiaceae warrant further investigation and that at least 1300 active antiplasmodial species are highly unlikely to be investigated by conventional approaches. While traditional and Indigenous knowledge remains vital to our understanding of people-plant relationships and an invaluable source of information, these results indicate a vast and relatively untapped source in the search for new plant-derived antiplasmodial compounds.

## 1 Introduction

Malaria is a life-threatening disease that affected 247 million people globally in 2021, with a disproportionately high number of cases (95%) occurring in Africa (WHO, 2022b). Although global case incidence, deaths and mortality rates for malaria have fallen over the past two decades, this downward trend has plateaued since 2015 and there were an estimated 619,000 malaria deaths in 2021 (WHO, 2022b). The two main treatments for the most prominent malaria-causing species, *Plasmodium falciparum* and *P. vivax*, are chloroquine and artemisinin-based combination therapies (involving artemisinin or derivatives). In 2008, due to chloroquine resistance, the World Health Organisation (WHO) recommended that *P. falciparum* infections should be treated with artemisinin-based combination therapies instead of chloroquine (WHO, 2008), but chloroquine resistance still persists (Ocan et al., 2019). Resistance to existing antimalarial drugs is an escalating challenge for eliminating malaria, indeed, there is concerning evidence of strains partially resistant to artemisinin emerging in Africa (Uwimana et al., 2020). As a result, the WHO recommends that research into antimalarial medicines should be accelerated as part of an effort to reach global malaria targets (WHO, 2022b).

Plants have provided or inspired the development of numerous pharmaceutical drugs (Howes et al., 2020; Newman and Cragg, 2020), including those on the WHO’s Model List of Essential Medicines (WHO et al., 2021). In the context of malaria, both chloroquine and artemisinin are derived from plants – chloroquine being a synthetic analogue of quinine, from *Cinchona* L. species (Rubiaceae: Gentianales) (Meshnick and Dobson, 2001) while artemisinin is extracted from sweet wormwood, *Artemisia annua* L. (Asteraceae: Asterales) (Qinghaosu Antimalaria Coordinating Research group, 1979). Furthermore, the antimalarial drug atovaquone was inspired by the chemical lapachol, which occurs in *Tabebuia* Gomes ex DC. species (Bignoniaceae: Lamiales) (Milliken et al., 2021). These are excellent examples of the natural solutions offered by plants and motivate the search for further plant-derived antimalarial drugs, particularly in the context of emerging resistance to existing antimalarials.

The predominant plant selection approach in the search for new antiplasmodial compounds has been an ethnobotanical one, that is, plants are investigated pharmacologically based on a history of traditional usage for malaria or other fever-causing diseases. This approach has provided some major successes, for example, the development of both quinine and artemisinin arose from traditional ethnobotanical knowledge (Qinghaosu Antimalaria Coordinating Research group, 1979; Meshnick and Dobson, 2001). However, this approach is restricted to a relatively small group of plant species and is limited in terms of reliability. It is therefore timely to assess whether emerging technologies, such as machine learning, could be used to more reliably harness the potential of plants as sources of new lead compounds for drug development.

**Figure 1:**
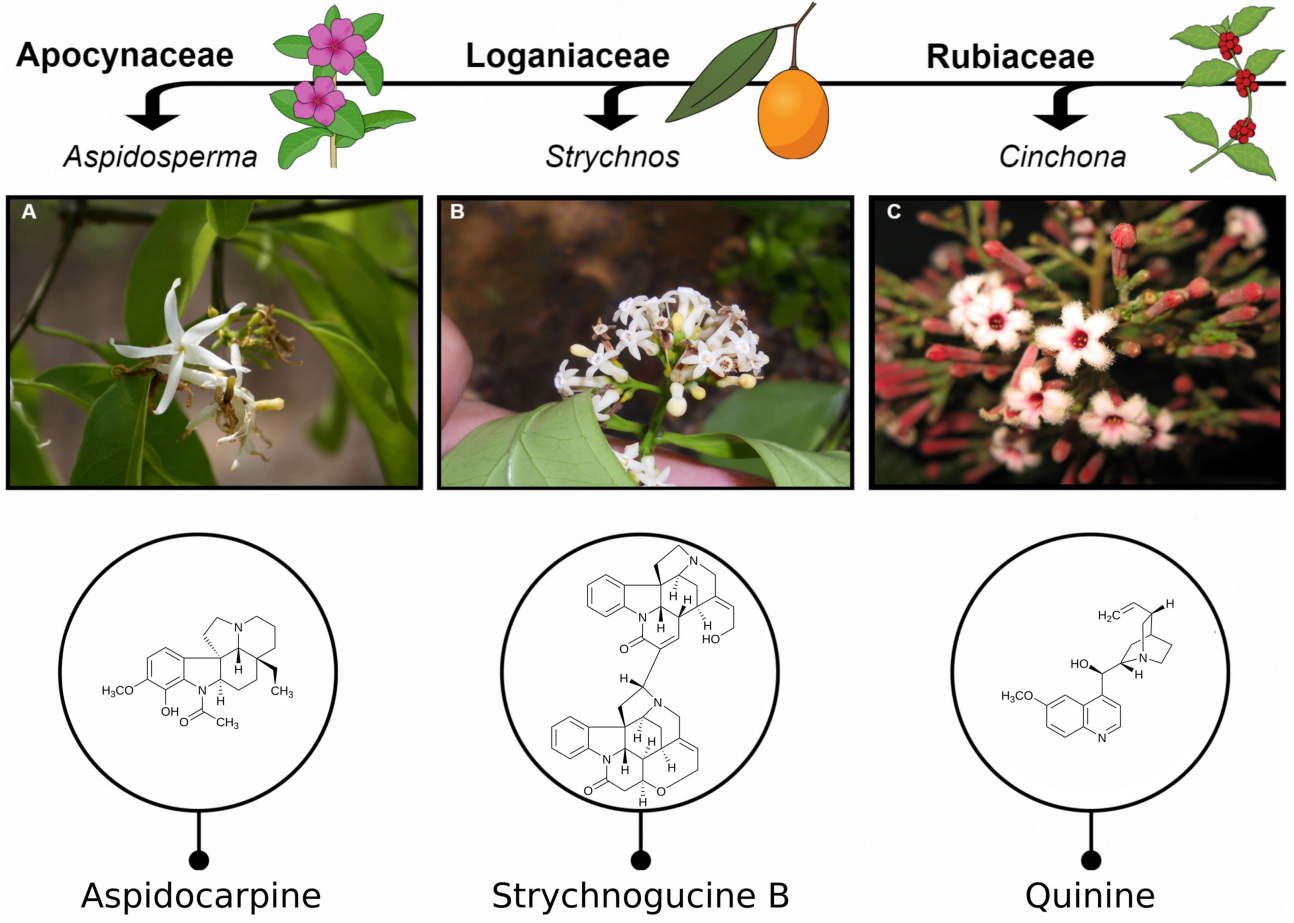
Examples of active antiplasmodial compounds in Apocynaceae, Loganiaceae and Rubiaceae. **(A)** Aspidocarpine from species in the genus *Aspidosperma* Mart. & Zucc. (Apocynaceae). **(B)** Strychnogucine B found in species of the highly diversified genus, *Strychnos* L. (Loganiaceae). **(C)** Quinine, the well-known antimalarial, found in the genus *Cinchona* L. (Rubiaceae). Photos by Cássia Bitencourt **(A)**, Lucas Marinho **(B)** and Alexandre Antonelli **(C)**.

Here we investigate the potential of three flowering plant families from the order Gentianales – Apocynaceae, Loganiaceae and Rubiaceae – selected based on numerous taxa being sources of chemically diverse alkaloids, a compound class of particular pharmaceutical relevance (Daley and Cordell, 2021). Some examples of antiplasmodial alkaloids from these families are given in **Figure 1**. Antiplasmodial activity in these families has been relatively well studied, in part due to the presence of the potent antiplasmodial alkaloids, quinine and the isomer quinidine, from the *Cinchona* genus. The phytochemistry of these families has also been relatively well studied, including numerous reports on the presence of alkaloids, for example, (Muhammad et al., 2003; Suksamrarn et al., 2003; Federici et al., 2009; Wong et al., 2011; Daley and Cordell, 2021). Furthermore, from an ethnobotanical perspective, these families contain many species which are used traditionally to treat malaria (Milliken et al., 2021).

Our first aim is to assess whether machine learning models can be trained on plant trait data to predict the antiplasmodial activity of plants. To achieve this, we present a dataset for the three study plant families, quantifying the known antiplasmodial activity of species as well as a broad range of potentially salient predictors of activity, which we will use to train and test machine learning models. We compare the performance of the machine learning models with two ethnobotanical approaches. Our second aim is to highlight the potential of plants to provide novel antiplasmodial compounds. We address this by using the collected data to estimate the number of active antiplasmodial species in the three families and we also explore methods for correcting existing data biases, in order to infer a clearer picture of antiplasmodial activity in Apocynaceae, Loganiaceae and Rubiaceae.

## 2 Materials and methods

### 2.1 Data collection

Here we use the term ‘trait’ in a broad sense that encompasses a variety of plant properties and characteristics. We collected a wide range of traits including morphological, biochemical, environmental and geographic features, along with abstract features relating to medicinal usage and common knowledge of plant species. In the following, we provide detail of each of the collected traits. A summary of the collected data and detail of the data collection methods is given in the **Supplementary Material**.

#### 2.1.1 Taxonomy

We extracted accepted names of all species of the three families according to the World Checklist of Vascular Plants (WCVP) V7 (Govaerts et al., 2021), totalling 21,111 species – 6,495, 496 and 14,120 from Apocynaceae, Loganiaceae and Rubiaceae respectively. We use *Genus* and *Family* names as categorical traits.

#### 2.1.2 Ethnobotanical data

Due to the documented link between traditional medicinal usage and bioactivity, evidenced in, for example, (Krettli, 2009), we collected binary traits documenting the presence and absence of known antimalarial usage (*Antimalarial Use*) and general medicinal usage (*Medicinal*). To compile these data we conducted a comprehensive literature review of medicinal usage in the three plant families, along with data provided by the Medicinal Plant Names Services (MPNS, 2022) and references to medicinal usage on the Plants of the World Online (POWO, 2022).

As an extension of the ethnobotanical data, we included binary traits to capture whether a plant is commonly known – which we approximated by recording the presence of a Wikipedia^1^ page (*Wiki Page*) and the existence of a common name (*Common Name*). The existence of Wikipedia pages for species is determined by searching all species, subspecies and varieties (and their synonyms). Common name data are compiled from a variety of sources, outlined in the **Supplementary Material**, with the majority of the data coming from MPNS and the United States Department of Agriculture Plants Database (USDA, 2022b).

#### 2.1.3 Phytochemistry

There is much evidence of the pharmacological and pharmaceutical importance of plant-derived alkaloids (Cordell et al., 2001; Dey et al., 2020; Howes et al., 2020; Daley and Cordell, 2021) and we have therefore collected binary traits on their presence/absence. These data were collected through a comprehensive literature review as well as metabolite data compiled from KNApSAcK (Afendi et al., 2012).

Though the coverage of the alkaloid data is relatively good (980 species with reported presence or absence), for the vast majority (97%) of these species, reports indicate a presence of alkaloids compared to 3% where alkaloids are absent. This may be the result of reporting bias, where publications are focused on species found to contain alkaloids and absences of alkaloids are not published. To assess the prevalence of the reporting bias, we contacted 11 authors of papers after the year 2000 that solely reported presences to ask if they had found any absences which they did not publish. We received responses from three authors detailing three species where alkaloids had been tested for and not found. Rather than being an issue of reporting bias, it may be the case that the vast majority of species in these families produce alkaloids. There is some evidence for this from studies testing large numbers of species for alkaloids where both presences and absences are reported e.g. (Soto-Sobenis et al., 2001). In either case, current data on the presence of alkaloids are relatively uninformative and so is not included in the following analysis. Instead, we use the collected data on alkaloids to catalogue which species have been tested for alkaloids. We use these data to create a binary trait (*Tested for Alkaloids*) which we use to analyse the relationship between phytochemical knowledge and knowledge of antiplasmodial activity.

An important plant trait indicating potent bioactivity is the degree to which a plant is toxic. As we aim to capture bioactivity in a broad sense, we compiled data on toxicity to any vertebrate and invertebrate animals. We have included this as a binary trait (*Poisonous*). Poison data were compiled from numerous sources detailing plants considered to be poisonous, outlined in the **Supplementary Material**, with the majority of the data coming from the LitTox resource (Royal Botanic Gardens, Kew, 2021).

#### 2.1.4 Morphology

As a major putative role of certain phytochemicals is to protect plants from herbivores (Maldonado et al., 2017), it is plausible that other defence mechanisms have a relation to bioactivity. Furthermore, certain biologically active compounds (e.g. some diterpene alkaloids) are biosynthesised in particular morphological structures (e.g. plant trichomes/hairs) of certain plants (Tomlinson et al., 2022). Here we assess the presence of emergences (hairs or spines) which we include as a binary trait (*Emergence*). Emergence data have been collated by Gentianales specialists, supplemented by the TRY plant trait database (Kattge et al., 2020) and POWO.

Another morphological trait we consider is plant life-form, which may correspond to occurrences of specific phytochemicals (de Almeida et al., 2005). To facilitate collection and coverage of morphological data, and as life-forms and presence of emergences are often well conserved within genera in these families, we include these traits by using the predominant state at the genus level. As multiple life-forms may appear within a single genus, the life-form data are one-hot encoded giving a set of binary traits (*herb*, *liana*, *succulent*, *shrub*, *subshrub*, *tree*). Life form data were initially retrieved from the WCVP and Flora do Brasil (Jardim Botânico do Rio de Janeiro, 2022), then reviewed and modified by Gentianales specialists.

#### 2.1.5 Geographic regions with malaria

To examine the relationship between prevalence of malaria in a given geographic area and the number of tested species, we collected data indicating which species are found in regions where malaria transmission occurs. We identified those regions from various sources, including the World Health Organization Database (WHO, 2022a) and the World Bank Development Indicators (The World Bank, 2022) (see **Supplementary Material** for full details). Regions indicated in these sources were then mapped onto the World Geographical Scheme for Recording Plant Distributions (Level 3) (Brummitt et al., 2001). We then used the WCVP distribution data to identify which species occur in these malarial regions (either native or introduced), assigned to each species as a binary trait *In Malarial Region*.

#### 2.1.6 Environmental

There is some evidence of environmental impacts on bioactive metabolite concentrations and diversity, for example, (Defossez et al., 2021). To characterise the environmental niche of species, we followed the methodology of Zu et al. (2021). We first extracted geographic occurrence records from the Global Biodiversity Information Facility (GBIF)^2^ for each species using the rgbif package (Chamberlain et al., 2022) in R. Occurrence data from GBIF contain many inconsistencies (Meyer et al., 2016). Initially we cleaned the data by removing: records collected before 1945, records with no given coordinates or impossible coordinates, records with coordinate uncertainty over 20km, records with rounded coordinates and records where the quantity of species occurrences (individual counts) is zero. Next, using the CoordinateCleaner package in R (Zizka et al., 2019) we removed: records with zero longitude or latitude, records with equal longitude and latitude, records outside reported country, records within country or province centroids, records in country capitals, records with institutional coordinates and records with GBIF Head Quarters coordinates. Finally, we discarded occurrences where species were reported to be outside of their native or introduced botanical regions according to the WCVP.

We quantified species’ environmental conditions using a set of 17 soil, climate, and topographic variables essential to plant survival, growth and reproduction. We extracted five soil traits (*nitrogen content*, *pH*, organic carbon stock (*ocs*), *water capacity*) from the SoilGrids database (Hengl et al., 2017; Poggio et al., 2021), which were averaged over a 30cm depth, as well as *soil depth* to bedrock. The eight bioclimatic traits we used were (*bio1*, *bio4*, *bio10*, *bio11*, *bio12*, *bio15*, *bio16*, *bio17*); representing temperature (mean annual, seasonality, daily mean of the warmest quarter, daily mean of the coldest quarter), precipitation (annual amount, seasonality, mean monthly amount of the wettest quarter, mean monthly amount of the driest quarter). These were extracted from the CHELSA database V2.1 (Karger et al., 2017; Karger et al., 2021). We also extracted the Köppen-Geiger climate classification (*kg mode*) from GloH2O (Beck et al., 2018). *Elevation* and *breakline elevation* were extracted from GMTED2010 (Danielson and Gesch, 2011) and *slope* was calculated from the elevation data using the terra package in R (Hijmans, 2022).

To match the resolution of the occurrence records, all environmental rasters were upscaled to 10 arc-minutes (c. 20 km) using the aggregate function of the terra package and environmental traits were extracted for each species occurrence using the extract function. For the continuous traits, median values were then calculated across all occurrences of each species and for the categorical variable *kg mode* the mode of all occurrences of each species was used. To capture coarse spatial information we also included median *latitude* and *longitude* for each species, calculated from the occurrence records.

#### 2.1.7 Classifying activity

To generate a comprehensive dataset of antiplasmodial activity, we conducted a thorough literature review for details of antiplasmodial tests in Apocynaceae, Loganiaceae and Rubiaceae and assigned activity labels to species based on the available reports of *in vitro* and *in vivo* studies. As with many biological datasets (Bender and Cortes-Ciriano, 2021), providing class labels is a nontrivial problem as there are many variations on the experiments and methods used for reporting activity. A detailed summary of the designated classification scheme we chose is given in the **Supplementary Material**. In general, for *in vitro* studies testing activity against *Plasmodium* parasites, the potency of IC50 values for crude extracts follows the definitions given in (Rasoanaivo et al., 2004a) i.e. < 10*μg*/*ml* is *active* and ≥ 10*μg*/*ml* is *inactive*. For tests of isolated compounds, according to the Medicines for Malaria Venture^3^ compounds with IC50 values < 1 µM are designated as active and of interest for further investigation, thus we use this threshold in our data. For fractions, we use a threshold of 5 *μg*/*ml*, which in general corresponds with published author decisions of activity categories. For *in vivo* studies, we use the published author decisions regarding activity.

#### 2.1.8 (Pseudo)absences

For some traits and datasets, presences are commonly reported but absences are not. For example, there are various datasets listing poisonous plants but published data on ‘safe’ plants are sparse. In many cases, this is likely a result of reporting bias, however there are multiple possible reasons for this. For certain traits there are presence biases e.g. in the case of poisons, once a plant has been found to be poisonous it can be reported as such; however if a plant is assessed for its toxicity, there are various caveats which limit the ability to confidently say the plant is safe. Examples of such caveats include the effect of extraction or preparation method on toxicity, the specific plant part tested, and which organisms the plant is toxic to. These variables exist in addition to methodological differences in assessing toxicity and also that *in vitro* studies may not correlate with effects *in vivo* (Houghton et al., 2007).

Where missing data give a strong indication of a genuine absence, i.e. for *Common Name*, *Poisonous*, *Medicinal*, *Wiki Page*, *Antimalarial Use*, *Emergence*, we take these pseudoabsences to be absences and fill missing values with 0. Missing values for other traits are left as NA and, where necessary, will be imputed.

### 2.2 Analysing and correcting sampling bias

An obstacle to our analysis is the significant sampling bias in the data. In part this has been created by the *ethnobotanical approach* to drug discovery. In this approach, researchers carry out (or rely on) ethnobotanical surveys that document traditional medicinal uses of plants. Plants used traditionally for malaria are then investigated to determine whether there is any scientific basis (e.g. antiplasmodial activity) that could explain the traditional use. As a result, plants traditionally used for malaria are significantly over-represented in the data on antiplasmodial activity of plant species.

In this section we outline the methods used to evidence the existence of the sampling biases as well as a method we use for correcting sampling bias, which may allow for a better picture of antiplasmodial activity and may be applied when training and evaluating machine learning models. Throughout this paper we use *labelled* to indicate species which have been classified as *Active* or *Inactive* following the scheme described in Section 2.1.7. We use *unlabelled* to indicate species with unknown antiplasmodial activity. The *underlying population* refers to all species in Apocynaceae, Loganiaceae and Rubiaceae.

Firstly, we compare the labelled data with the underlying population by highlighting common choices made by researchers when selecting plants to test for antiplasmodial activity. We then statistically verify the differences using the Chi-squared test (Pearson, 1900) for the discrete traits and the Kolmogorov–Smirnov 2-Sample test (Smirnov, 1939) for the continuous traits. In order to account for the repetition of multiple tests and the associated family-wise error rate, we adjust the significance thresholds using the Holm-Bonferroni method (Holm, 1979).

Before describing the bias correction method we have implemented, we first outline our assumptions about the nature of the bias. Let *s* be a binary variable denoting the sampling decision i.e. 1 indicates a sample is in the labelled data and 0 indicates a sample is unlabelled. Given a species with traits *x* and activity label *y*, we assume that the sampling decision, P(s|x,y) , is independent of *y* given *x*, P(s|x,y)=P(s|x) i.e. plants are tested without *a priori* knowledge of their activity, *y*, but based on traits, *x*, that might increase the probability of active compounds compared to random sampling. This is commonly known as the missing at random (MAR) assumption (Zadrozny, 2004).

As described by Cortes et al. (2008), we can correct for sampling bias by reweighting the sampled (labelled) data using the inverse of the sampling probability for each sample, 1/P(s|x) ^4^, a technique often referred to as Inverse Probability Weighting. Under this procedure, the reweighted data will resemble the underlying population if P(s|x) is accurately estimated. As an example in the context of the current study, species which are traditionally used for malaria have a relatively high probability of being tested and as a result are over-represented in the available sample i.e. P(s|x) is large for these species and so the assigned weight is small.

To predict P(s|x) , we use a regularised Logistic Regression model, implemented in the scikit-learn Python library (Pedregosa et al., 2011) which we refer to as the Correction Model. We use such a model to limit overfitting and as Logistic Regression models are generally well calibrated. Given a sample (labelled) dataset and underlying population, instances in the sample dataset are labelled *s* = 1 and instances not in the sample are labelled *s* = 0. The Correction Model is trained to predict *s* from the given traits such that, assuming good calibration, the probability estimates given by the model correspond to P(s|x) .

Prior to training the model, the categorical traits *Genus*, *Family* and *kg mode* are target encoded in the preprocessing step using the category_encoders library (Micci-Barreca, 2001). The traits are then scaled by removing the mean and scaling to unit variance. Finally, we use the scikit-learn (Pedregosa et al., 2011) k-Nearest Neighbor imputer to impute any missing values. Missing values of a trait from a given sample are imputed by assigning the mean trait value of the five samples nearest to the given sample, where nearness between two samples is measured with the Euclidean distance using the traits that neither sample is missing.

To verify the accuracy of this bias correction approach, we calculated the mean Brier score (Brier, 1950) of the predicted probabilities in 10 iterations of 10-fold stratified cross validation. The Brier Score measures the difference between the predicted probability given by the model and the actual label (*s* = 0 or 1). We also visualise the accuracy of the bias correction approach by comparing the means of the traits in the labelled data, underlying population and the bias-corrected labelled data.

### 2.3 Machine learning models

To explore the success of different plant selection approaches and motivate a machine learning based approach to the problem, we train Support Vector (SVC), Logistic Regression (Logit) (Pedregosa et al., 2011), XGBoost (XGB) (Chen and Guestrin, 2016) and Bayesian Neural Network (BNN) (Silvestro and Andermann, 2020) classifiers and compare these with two ethnobotanical approaches: selection based on traditional antimalarial use and selection based on traditional medicinal use not specific for malaria.

As the cost of false positives is relatively high – resources will be misallocated in trying to find antiplasmodial compounds in inactive species – we aim to maximise *precision* of the models i.e. the proportion of species which are predicted to be active that are correctly predicted. Of course, *recall* (the proportion of active species predicted to be active) is still important as a large list of antiplasmodial species provides more opportunities for finding new antiplasmodial compounds. However, even with very low recall the models will still generate very large lists of antiplasmodial species from the 21,111 species in Apocynaceae, Loganiaceae and Rubiaceae. As a result, we aim to maximise the F-score with *β* = 0.5 (*F*_0.5_), i.e. the harmonic mean of precision and recall with more importance given to precision. We evaluate the models with this score along with precision, and also provide precision-recall curves.

We evaluate the models using 10 iterations of 10-fold stratified cross validation in two settings. Firstly, we analyse model performance in the usual case, where the models are trained and tested on folds of the given data. We also attempt to estimate model performance on the underlying population by assigning sample weights to the labelled data, using the method discussed in Section 2.2, such that the given labelled data is more representative of the underlying population. In this case, sample weights are used in both training and testing.

#### 2.3.1 Preprocessing

In the preprocessing step, the categorical traits *Genus*, *Family* and *kg mode* are target encoded. The traits are then scaled by removing the mean and scaling to unit variance. We then use the scikit-learn (Pedregosa et al., 2011) k-Nearest Neighbor imputer, trained using the training data and the unlabelled data, to impute any missing values. Finally, we use Principal Component Analysis (PCA), implemented in scikit-learn, to reduce the dimensionality of the highly colinear continuous environmental traits. The PCA is trained using the training data and unlabelled data and the number of components used in the PCA is selected such that at least 80% of the variance is explained by the components. The traits *In Malarial Region* and *Tested for Alkaloids* were collected for the analysis of sampling bias rather than as predictive traits and so are not included in the machine learning models.

#### 2.3.2 Training

The Logit, SVC and XGB classifiers are trained as follows. Given a set of training folds and a test fold, hyperparameters of the models are tuned via cross validation on the training data using GridSearchCV (Pedregosa et al., 2011). In this step, *F*_0.5_ is used as the evaluation metric and we tune a basic list of hyperparameters in order to minimise under/overfitting and to maximise *F*_0.5_. For the Logit and SVC classifiers, we tune the regularisation parameter C, as well as the class_weight parameter. For the XGB classifier, we tune the max_depth parameter. Once the best hyperparameters for the models have been generated the models are retrained on all the given training data (with/without sample weights depending on the evaluation setting).

For the BNN classifier, we use two layers of 10 and 5 nodes, respectively, and tanh activation function. We train the model through 100,000 Markov chain Monte Carlo iterations, as implemented in npBNN (Silvestro and Andermann, 2020), with/without sample weights depending on the evaluation setting. We use 1,000 posterior samples of the parameters when generating predictions.

### 2.4 Assessing activity in the study families

In order to motivate further exploration of these three plant families as potential sources of new pharmaceuticals, we use the collected data to estimate the antiplasmodial activity of the families in two ways. Firstly, we summarise the proportion of active species in each family using the collected labelled data. As this is likely to be unrepresentative due to the sampling biases, we also provide a summary of the labelled data when the bias is corrected using the method discussed in Section 2.2.

We also use the estimation of P(s|x) , discussed in Section 2.2, to analyse the existing sampling decision and highlight the wealth of potentially active species that are currently overlooked. First, we compare P(s|x) for the known active and inactive species in the labelled data. We then analyse species that are highly unlikely to be tested according to the existing sampling decision and we take these to be species for which P(s|x) is below the median value in the unlabelled data. We check the known activity of these species and use the machine learning model with the highest precision to provide a conservative estimate of how many of these species are active in the underlying population. The estimate of the number of active species given by the model is corrected using an estimate of the model precision. The model precision estimate is generated from the mean precision given in the cross-validation evaluation and we calculate a 95% bootstrap confidence interval from the precision scores given in each fold of the cross-validation.

## 3 Results

### 3.1 Data summary

#### 3.1.1 Labelled data

Following the scheme for classifying activity described in Section 2.1.7, we designated 132 species as active and 150 species as inactive, providing 282 labelled species from the 21,111 species in Apocynaceae, Loganiaceae and Rubiaceae. In these labelled data, all species are given trait values for each of the traits except for those trait values which rely on GBIF occurrence records where data are missing for five species. 

#### 3.1.2 Trait relations

**Figure 2:**
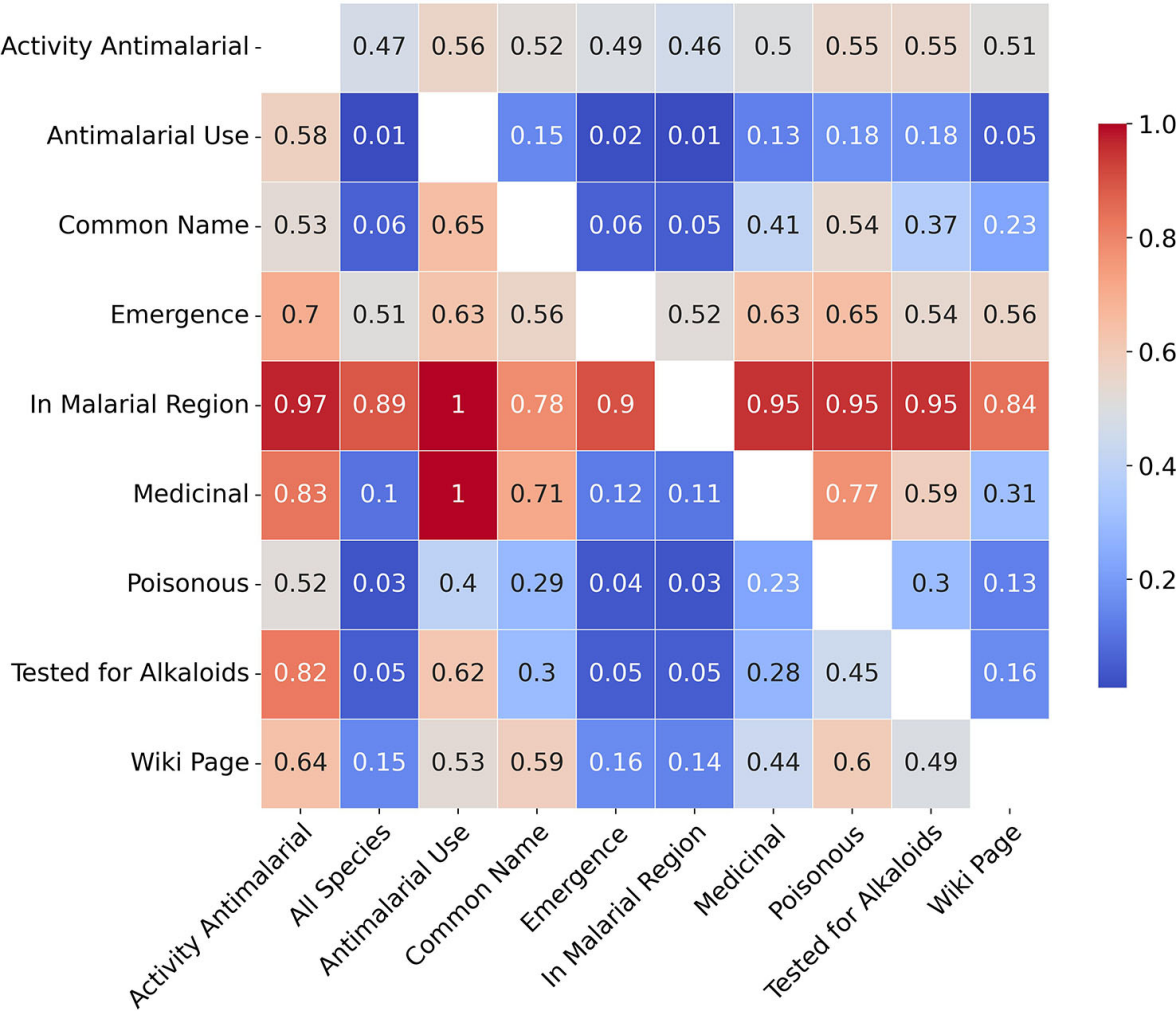
Co-occurrence heatmap summarising collected binary traits.

**Figure 2** provides a brief overview of the collected data, summarising relationships between some of the traits from all the collected data. The heatmap gives a visualisation of the co-occurrences of the binary traits, and the given values correspond to the mean values of traits in the *y* axis when traits in the *x* axis are present, while ‘All Species’ provides a comparison with mean values of traits in the underlying population. For example, 1% of all species are used traditionally for malaria while 18% of poisonous species are used traditionally for malaria. Similarly 10% of all species are used as traditional medicines while 77% of poisonous species are used as medicines. With regards to activity, the first column provides mean trait values for active species which indicate stark differences with the underlying population (e.g. 52% of active species are poisonous compared to 3% in the underlying population). However, these differences are more a reflection of the sampling biases rather than any strong relationships between the traits and antiplasmodial activity.

### 3.2 Sampling bias

#### 3.2.1 Comparing the labelled data with the underlying population

The most common feature motivating the selection of plants to test for antiplasmodial activity is traditional knowledge of use for malaria, for example (Andrade-Neto et al., 2003; Bourdy et al., 2004; Bertania et al., 2005; Ramalhete et al., 2008; Ezike et al., 2016; Taek et al., 2021). We found that 48% of labelled species are traditionally used for malaria while only 1% of species in the underlying population are traditionally used for malaria. Similarly, plants are frequently tested based on more general traditional medicinal usage (not specific to malaria), e.g. (Kaushik et al., 2013; Mothana et al., 2014; Singh et al., 2015; Satish et al., 2017). 77% of labelled species are traditionally used as medicines while 10% of species in the underlying population are traditionally used as medicines.

As previous successes in finding plants with antiplasmodial activity have linked their alkaloid content to the antiplasmodial activity, tests of antiplasmodial activity are often conducted on plants known/expected to contain alkaloids. For example (Wright et al., 1992; Solis et al., 1995; Likhitwitayawuid et al., 1999; Weniger et al., 2001; Mitaine-Offer et al., 2002; Federici et al., 2009). Moreover, in many reports where plants are tested for antiplasmodial activity, those studies also include tests for (and find) alkaloids e.g. (Likhitwitayawuid et al., 1999; Muhammad et al., 2003; Suksamrarn et al., 2003; Wong et al., 2011). As a result, 69% of labelled species and 82% of active species have been tested for presence of alkaloids, while only 5% of species in the underlying population have been tested for presence of alkaloids.

Another potential factor influencing sampling is the geographic location of species, i.e. plants occurring in regions with malaria are commonly selected to test for antiplasmodial activity, for example (Rasoanaivo et al., 2004b; Bertania et al., 2005; Al-Musayeib et al., 2012; Kantamreddi and Wright, 2012; Taek et al., 2021). As a result, 99% of labelled species are found in malarial regions compared to 89% in the underlying population. In fact, there is only one tested species which is not found in a malarial region (*Gardenia urvillei* Montrouz. (Rubiaceae) which is native to New Caledonia) and three *Ochrosia* Juss. (Apocynaceae) species (native to Fiji, Tonga and New Caledonia) whose activity is known through the presence of antiplasmodial compounds (not themselves explicitly tested) which are not found in malarial regions.

It is also common to test plants taxonomically related to known antiplasmodial plants (Weenen et al., 1990; Frédérich et al., 2002; Philippe et al., 2005; dos Santos Torres et al., 2013; Brandão et al., 2020). For example, some genera known to contain active species are frequently tested e.g. *Aspidosperma* (Apocynaceae: Gentianales) (18 labelled species) and *Strychnos* (Loganiaceae: Gentianales) (36 labelled species).

For almost all the quantitative traits, the difference between the labelled data and underlying population (as measured by Chi-squared test for the discrete traits and the Kolmogorov–Smirnov 2-Sample test for the continuous traits) is significant (corrected p values < 0.05) with the exception of life-forms (lianas and succulents). The most diverging traits are *Antimalarial Use* and *Tested for Alkaloids* (corrected p values = 0, Chi-squared statistic 3137 and 2218 respectively). We can therefore conclude that the labelled data significantly differ from the underlying population. Overall, it is apparent that the approaches used to select plants for antiplasmodial tests have biased the available data on antiplasmodial activity.

#### 3.2.2 Bias correction

**Figure 3:**
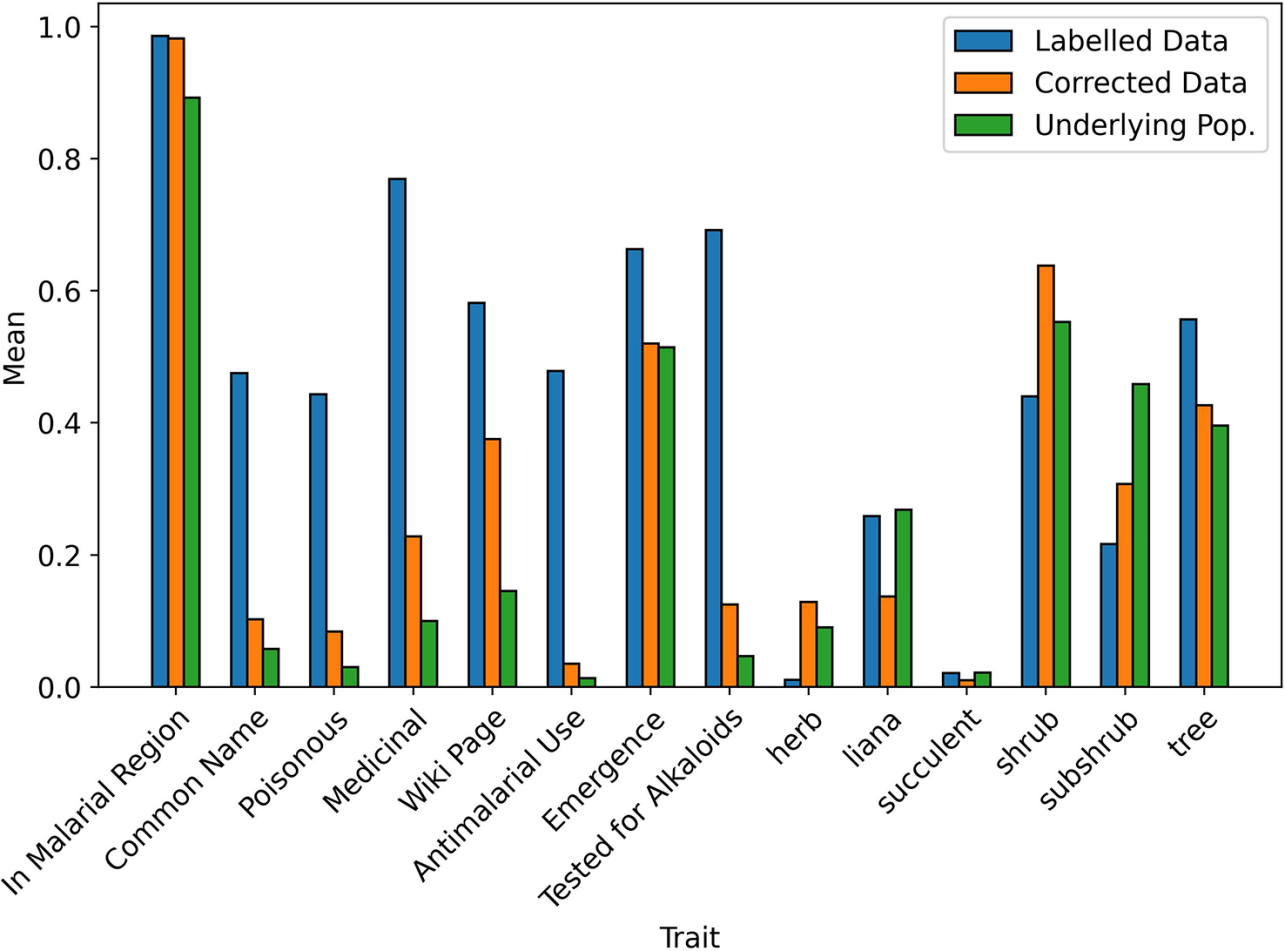
Mean values of binary traits in biased and corrected datasets.

**Figure 4:**
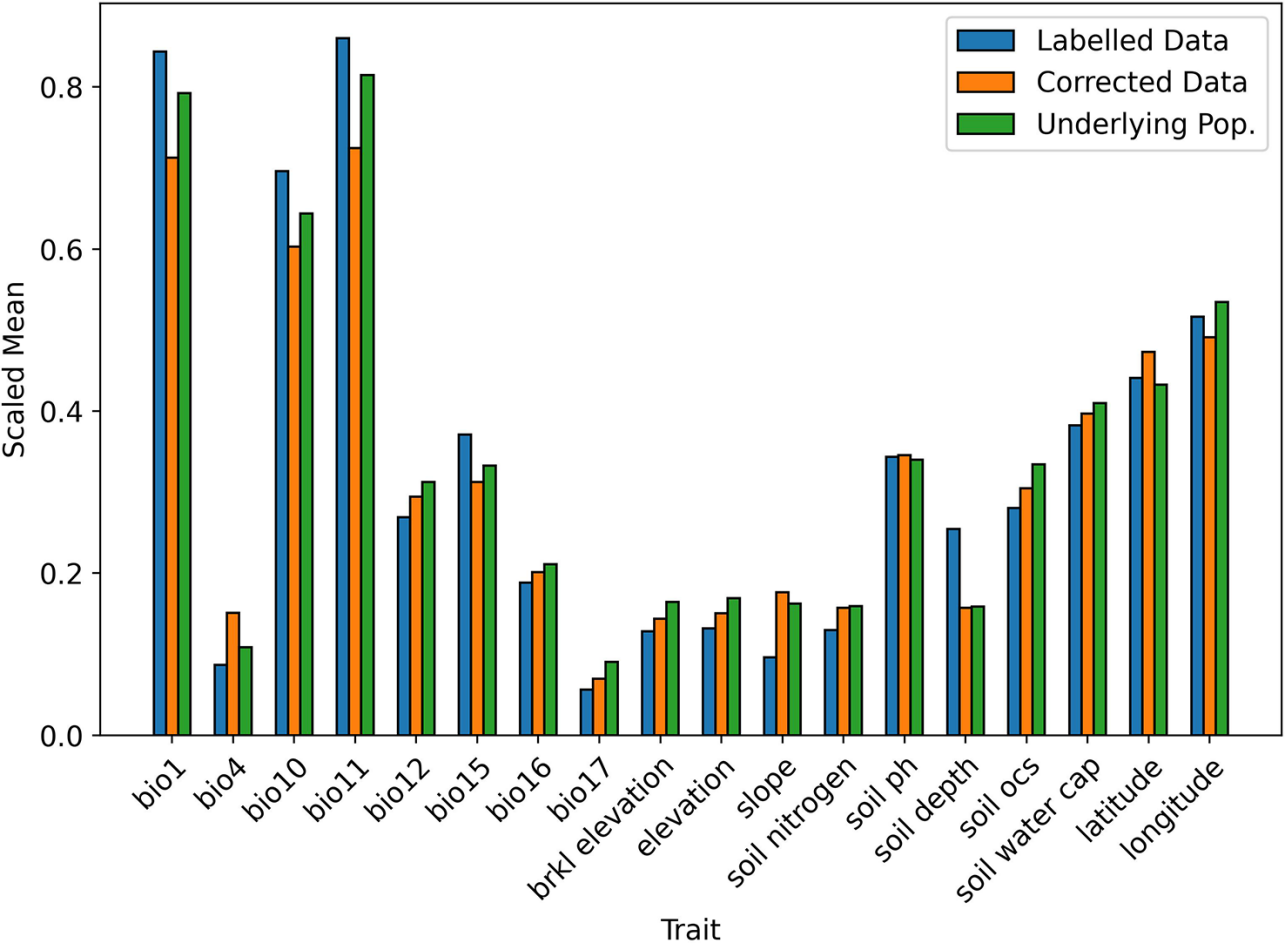
Scaled mean values of continuous traits in biased and corrected datasets.

When testing the Correction Model in 10 iterations of 10-fold stratified cross validation, the mean Brier score was 0.0097 (SD = 0.001), indicating an accurate fit to the data and so, a reliable prediction of the selection probability. A visual comparison of the bias-corrected data and the underlying population is given in **Figures 3**, **4**. For readability, the mean values of the continuous traits are rescaled between 0 and 1 using the MinMaxScaler from scikit-learn (Pedregosa et al., 2011). We can see that for the majority of the traits, the mean values of the corrected data closely resemble the underlying population compared to the values in the labelled data.

### 3.3 Comparing plant selection approaches

Given the quantification of antiplasmodial activity, we may now analyse the effectiveness of different approaches for plant selection – random selection, selection based on traditional antimalarial use (Ethno (M)) and selection based on general traditional medicinal use not specific for malaria (Ethno (G)). **Table 1** provides a summary of the precisions of these methods on the biased and corrected datasets. When plants are selected based on a history of use for malaria or general medicinal usage, they are more likely to be active than selecting plants at random (both in the biased and corrected cases). This result provides some validation for the ethnopharmacological approach and agrees with the findings of (Krettli et al., 2001). However, in Apocynaceae, Loganiaceae and Rubiaceae, only 281 species have a history of antimalarial usage and 2109 have a history of general medicinal usage which limits the search for new compounds to a relatively small group of plants.

**Table 1:** Precision of selection strategies.

	Uncorrected	Corrected
**Random**	0.47	0.36
**Ethno (G)**	0.50	0.42
**Ethno (M)**	0.56	0.42

Considering the sampling decision more generally, in the uncorrected case, the value for the ‘Random’ approach reflects the mean activity of all tested species and provides some quantification of the overall precision of the existing plant selection approach i.e. species selected for testing by researchers have a probability of being active of 0.47, while the estimate of the mean activity of the underlying population is 0.36. Similarly, the mean value of P(s|x,y) for active species in the labelled dataset is 0.53, while for inactive species this value is 0.31.

#### 3.3.1 Machine learning evaluation

##### 3.3.1.1 Without bias correction

**Figure 5:**
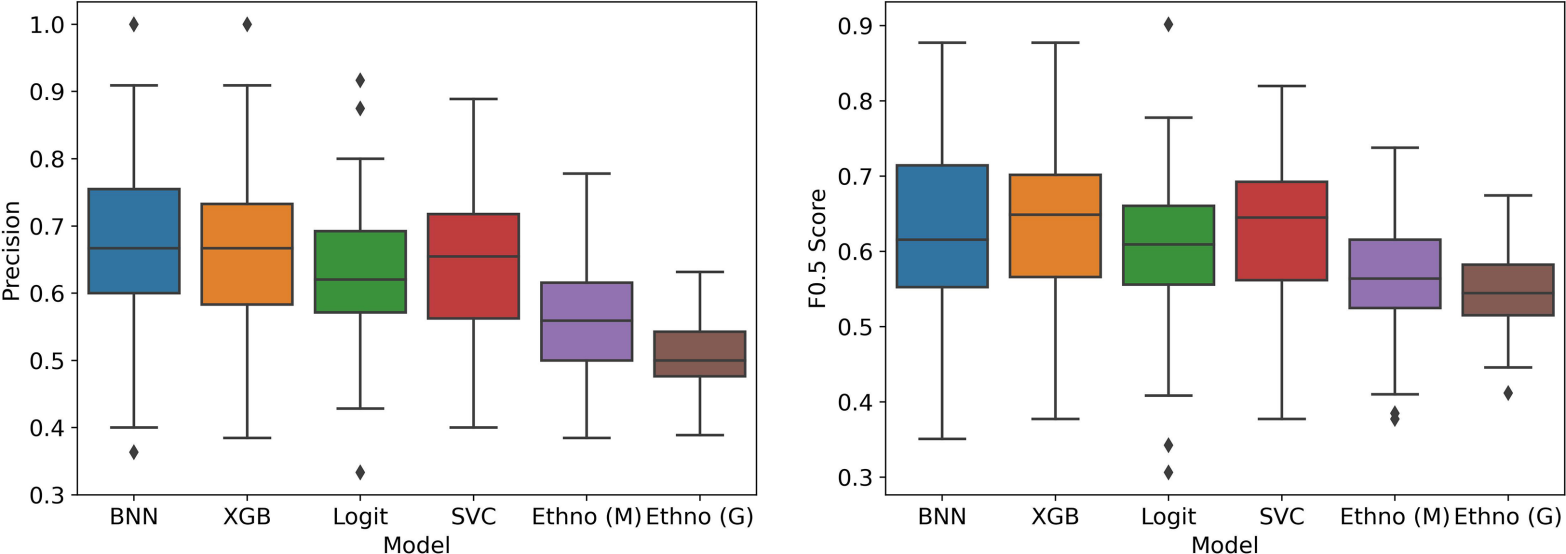
Model performance in stratified cross validation without bias correction.

**Figure 6:**
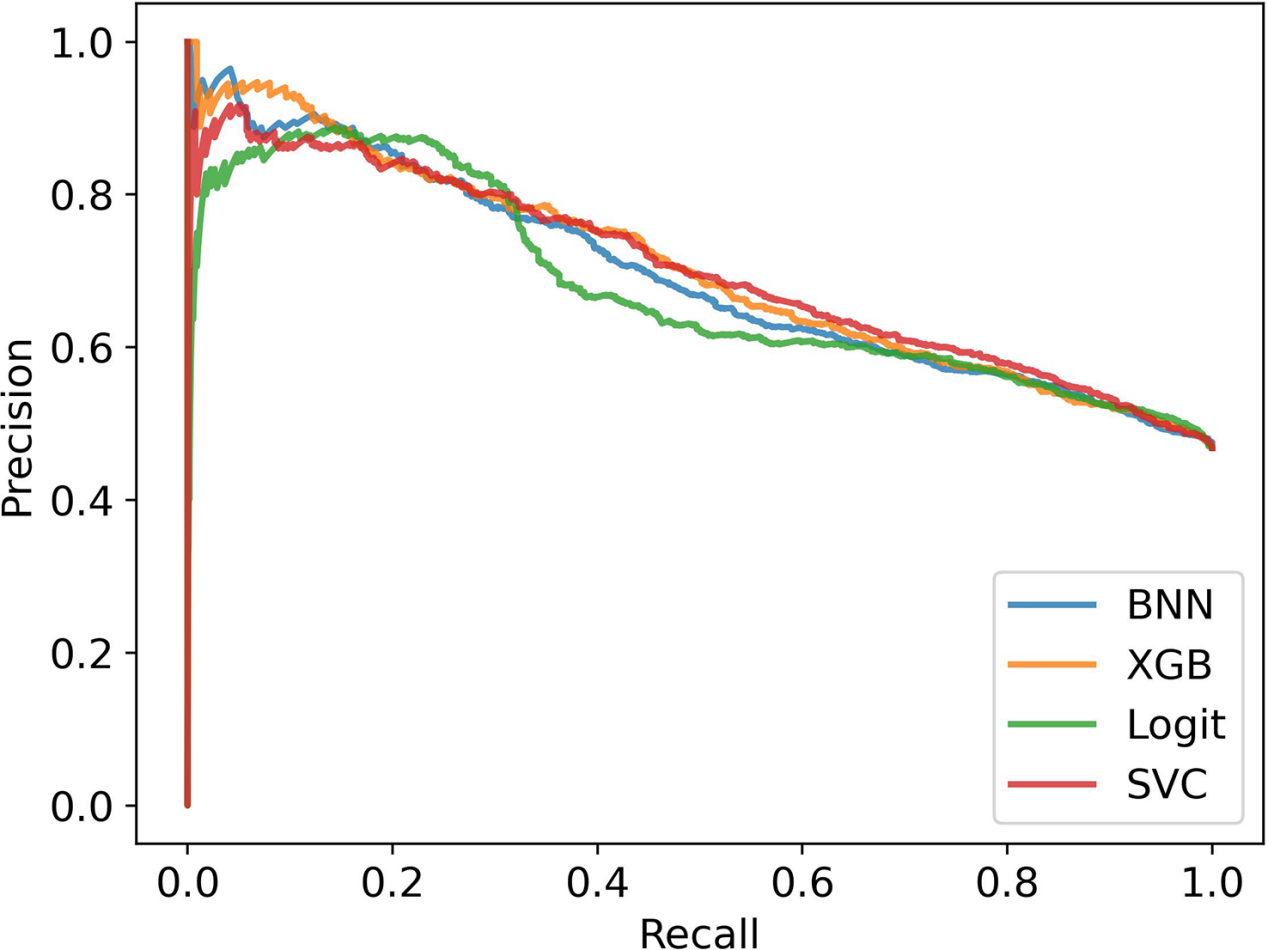
Precision-Recall Curves in stratified cross validation without bias correction.

In **Figure 5**, we see the performance of the machine learning models compared to the two ethnobotanical approaches. Overall the mean scores of the machine learning models improve on both approaches and indicate that antiplasmodial activity can be predicted relatively accurately from the collected traits (mean precisions – BNN: 0.66, XGB: 0.66, Logit: 0.62, SVC: 0.65, Ethno (M): 0.57, Ethno (G): 0.50). The Precision-Recall curves in **Figure 6**, generated using all test instances in the cross validation, show how varying the classifier thresholds can improve precision at the cost of recall, for example, by increasing the threshold of the models we can achieve a precision of over 0.8 with a recall of approximately 0.2.

##### 3.3.1.2 Corrected performance

**Figure 7:**
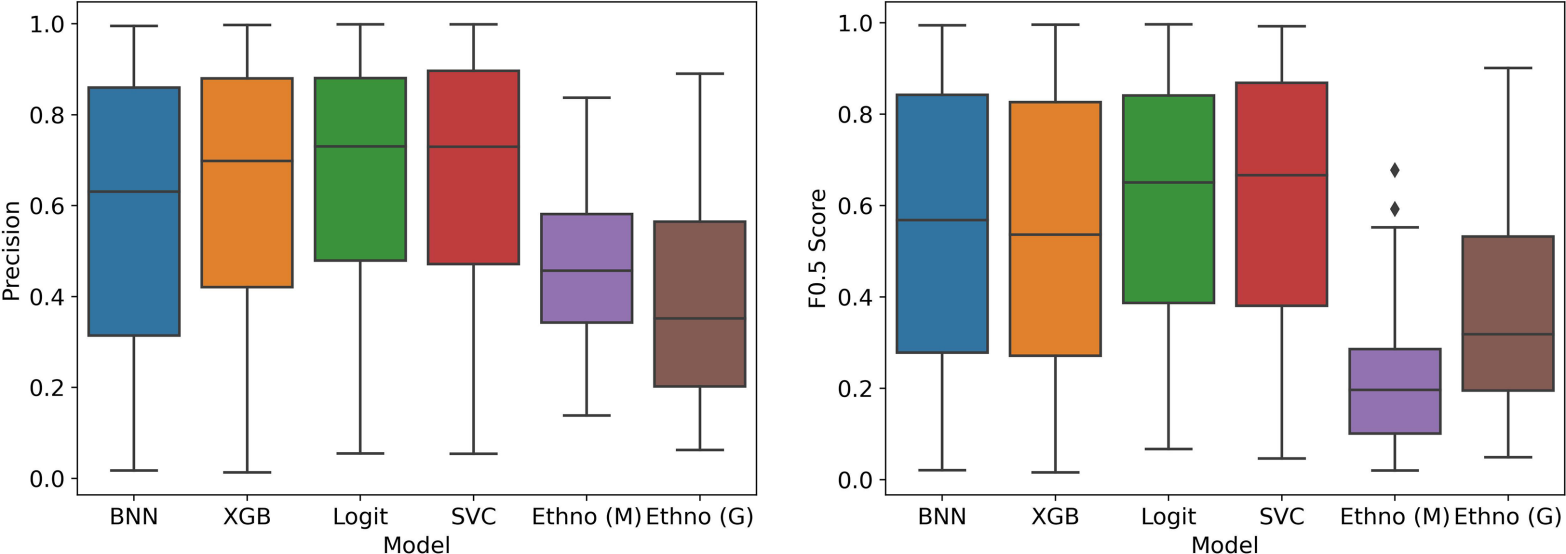
Model performance in stratified cross validation with bias correction of training and testing samples.

**Figure 8:**
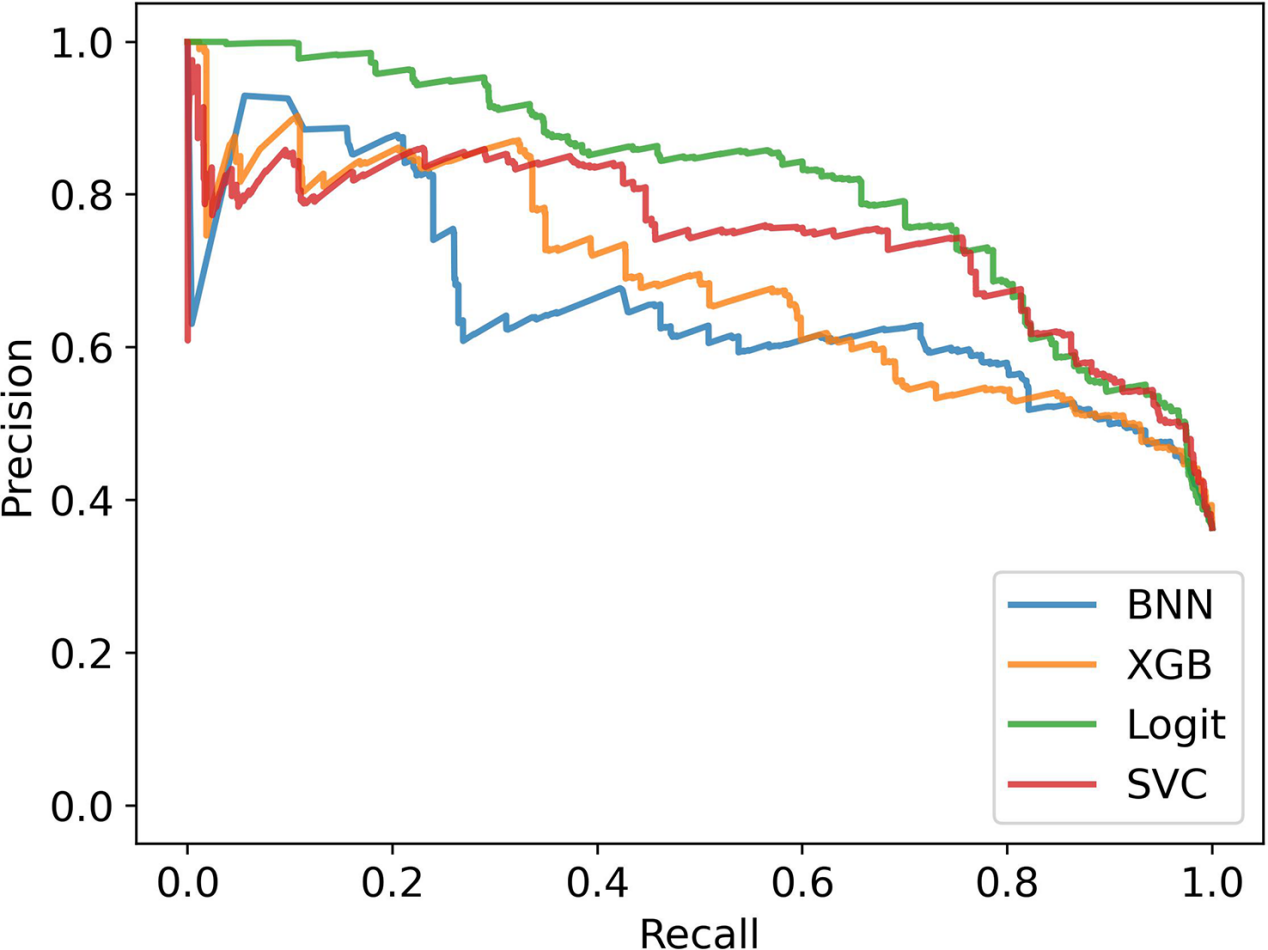
Precision-Recall Curves in stratified cross validation with bias correction of training and testing samples.

**Figures 7**, **8** show the estimated performance of the models on the underlying population. Again, though there is higher variance in model performance due to the weights used on the train and test samples, the machine learning models improve on the ethnobotanical approaches. Moreover, above we estimated that the precision of the existing plant selection approach of the field as a whole was 0.47, and our models again compare well with this (mean precisions – BNN: 0.59, XGB: 0.63, Logit: 0.66, SVC: 0.67).

### 3.4 Antiplasmodial potential of Apocynaceae, Loganiaceae and Rubiaceae

In **Table 2**, we provide a summary of the proportion of active species in each family. The given labelled data suggest a high level of activity in the families (47%), though when we estimate the activity of the underlying population by correcting for the sampling biases, the proportion is lower (36%). Nevertheless, this estimate indicates that there are approximately 7677 species in these families that may warrant further investigation.

**Table 2:** Estimated proportions of active species.

	Uncorrected	Corrected
**Apocynaceae**	0.57	0.51
**Loganiaceae**	0.30	0.12
**Rubiaceae**	0.41	0.34
**All**	0.47	0.36

#### 3.4.1 Surprises

For those species that we deem highly unlikely to be tested, ( P(s|x)<0.0014 ) only 2 such species are in the labelled data where one is known to be active, while 9997 are in the unlabelled data. When the SVC model is trained on all the available data and used to predict the activity of these species in the unlabelled data, 2358 are estimated to be active. This gives a 95% confidence interval of 1300 – 1522 active species when the model precision is accounted for. Note that this is a conservative approximation as we are only considering species that the model predicts to be active and correcting for the estimated false positives. However, as visible in the Precision-Recall curves, recall of the models is not perfect and it is highly likely that there are also a significant number of species that the model predicts to be inactive species which are in fact active.

## 4 Discussion

In this study we have shown that machine learning models based on plant traits can be effective at selecting active antiplasmodial plants. Moreover, as the machine learning models output a classification confidence for each sample, researchers searching for active species may select samples which are labelled as active with most confidence by the models. The Precision-Recall curves in Section 3.3.1 indicate that such an approach could yield a large number of active species with a precision of at least 0.8.

We have also extensively considered sampling biases in the data, an issue that exists in botany (Meyer et al., 2016; Visscher et al., 2022) and biological sciences more generally (Bender and Cortes-Ciriano, 2021). We have used a bias correction method to provide a more accurate representation of the properties of the underlying data and a more robust evaluation of plant selection methods. We hope that by tackling sampling bias in our particular context we raise awareness of this issue in botany more widely and highlight potential solutions to this problem.

Our results suggest that there are a large number of species (approximately 7677) in Apocynaceae, Loganiaceae and Rubiaceae with antiplasmodial potential while only 281 species have a history of antimalarial usage. Furthermore, of those species we deem highly unlikely to be investigated, we estimate at least 1300 untested species to be active. These results indicate a vast and relatively untapped source to accelerate the search for new plant-derived antiplasmodial compounds.

We have so far explored the potential of machine learning in predicting antiplasmodial activity. However, activity is not the only metric to evaluate useful medicinal plants. For example, useful active compounds found in plants will ideally also be more selective for *Plasmodium* parasites and less toxic to human cells. Plants used traditionally as oral preparations, which have a long history of use, may give some indication of their safety and/or possible selectivity, which is a potential benefit of selecting traditionally used plants. Moreover, our machine learning approach does not yet provide any indication of which plant parts contain the active compounds, and which extraction methods optimise their concentrations; in contrast to traditional preparations that specify plant parts and methods for their preparation. Nevertheless, finding active antiplasmodial plants is still a critical step in the search for new antiplasmodial plant-derived compounds with potential lead structures/pharmacophores to facilitate future drug discovery for malaria. The urgent need to find new antimalarial drugs exists against a backdrop of escalating resistance to existing antimalarial drugs (Uwimana et al., 2020), and in the context that the WHO’s Global Technical Strategy for Malaria (2016 – 2030) aims to ensure universal access to malaria prevention, diagnosis and treatment, an aim that is supported through harnessing innovation and expanding research (WHO, 2017).

In summary, we show that trait data-based machine learning models can outperform existing ethnobotanical plant selection approaches to find species with antiplasmodial activity, and provide a novel approach underpinning future work to predict the bioactivity of plant species. Plants are a known source of lead compounds for pharmaceutical drug development (Howes et al., 2020; Newman and Cragg, 2020) and more strategic and efficient approaches are needed to facilitate future drug discovery, particularly considering that there are an estimated 343,000 known vascular plant species (Govaerts et al., 2021) that remain largely unexplored scientifically. This study highlights the potential of integrating ethnobotanical knowledge with technological advances. While such integration creates promising opportunities, we stress the need that any material and non-material benefits are shared fairly and equitably with knowledge holders and stewards of plant diversity around the world (Antonelli, 2023). By exploring sustainable uses of biodiversity, societies are more likely to reach the ambitious goals and targets set under the recently established Kunming-Montreal Global Biodiversity Framework.

### 4.1 Related work and novelty

In this paper we have presented and evaluated a novel approach based on plant traits to predict the antiplasmodial activity of plants. Though there is some related work, e.g. predicting antiplasmodial activity of compounds (Egieyeh et al., 2018; Danishuddin et al., 2019; Bosc et al., 2021), predicting potential antiplasmodial plants using traditional antimalarial *usage* as a proxy (Pellicer et al., 2018; Milliken et al., 2021), predicting other related measures of bioactivity (Rønsted et al., 2012; Maldonado et al., 2017; Holzmeyer et al., 2020); we believe ours is the first to predict antiplasmodial activity of plants directly based on a combination of plant trait data.

In order to predict the antiplasmodial activity of plants, we have generated a comprehensive resource of plant traits and documented antiplasmodial activity for plants in the Apocynaceae, Loganiaceae and Rubiaceae families. With regards to antiplasmodial activity, the closest available datasets we were able to find detailing antiplasmodial plants were the metabolite and biological activity data from KNApSAcK (Afendi et al., 2012) and Dr. Duke’s Phytochemical and Ethnobotanical Databases (DPED) (USDA, 2022a). In an attempt to utilise the KNApSAcK data, we extracted information on known antiplasmodial metabolites from KNApSAcK and using the KNApSAcK database, were able to match these to plants which contain these compounds. Similarly, we downloaded the list of antiplasmodial plants in DPED and filtered the results to the study families. We found these data to be limited. Firstly, in both cases, the data are limited to antiplasmodial activity of specific compounds rather than antiplasmodial fractions or extracts from plants. Secondly, the coverage of the data is poor (from KNApSAcK: one active species in Apocynaceae, one in Loganiaceae and four in Rubiaceae; from DPED: 19 active species in Apocynaceae, one in Loganiaceae and ten in Rubiaceae). Also, though KNApSAcK and DPED provide references to the original research, it is not clear exactly what criteria are used to determine when a compound is an active antiplasmodial and in DPED many of the cases of ‘active’ species were due to presence of compounds with weak activity (e.g. lupeol, rutin, quercetin and betulinic acid). Finally, from these kind of data, it is difficult to ascertain with confidence which plants are inactive.

### 4.2 Future work

We have shown that the collected trait data can be used to predict antiplasmodial activity with machine learning approaches and basic preprocessing steps. However, though we have used a bias correction method to improve evaluation of the plant selection approaches, we recognise that the models must be tested on the underlying population in order to obtain a true measure of model performance. We hope to address this in future work by using the machine learning models to predict active species in the underlying population and assessing the activity of these predicted species in new antiplasmodial assays. Regarding training of the models, as we have seen, the antiplasmodial activity is known for only 282 species, resulting in a relatively small dataset for training machine learning models. We believe that small improvements in the existing data could further improve performance of the machine learning approaches, and, where possible, we therefore encourage further testing of species that are currently underrepresented in the existing data.

## Data availability statement

The original contributions presented in the study are included in the article/**Supplementary Material**. All finalised trait data and analysis are archived in https://doi.org/10.5281/zenodo.7836732. Further inquiries can be directed to the corresponding author.

## Author contributions

M-JRH, OP-E, EL, JR and AA conceptualized the study. AR-B, CB, DG, EL, M-JRH, CA, DR, IO and SP collated data and provided specialist input on datasets. DS provided specialist input on the machine learning methodology. CW provided specialist input on antiplasmodial activity. AR-B collated data, conducted analyses and drafted the original manuscript. All authors participated in writing and giving feedback on the manuscript. All authors have read and approved the final manuscript.

## Footnotes

1
https://www.wikipedia.org/ accessed on 14 Apr. 2022

2
https://www.gbif.org/what-is-gbif

3
https://www.mmv.org/20th-call-proposals accessed on 30 Aug. 2022.

4 The constant *P* (*s* = 1) is omitted.
