# Supplementary material for: Machine learning enhances prediction of plants as potential sources of antimalarials
Source: Front Plant Sci. 2023 May 25;14:1173328. doi: 10.3389/fpls.2023.1173328 (PMC10248027; doi:10.3389/fpls.2023.1173328)
Supplement: Supplementary file 2 [file DataSheet_2.pdf]

Supplementary Material

1 COMPILED DATA

Collected traits for Apocynaceae, Rubiaceae and Loganiaceae are given in separate repositories.[1,2] Where we have relied on literature reviews to collect data, appropriate references can be found in the manually collected data sections of the trait repositories for each family. All finalised trait data and analyses are given in the trait modelling repository.[3] The following traits were collected and used in the analysis (* denotes traits not used to train the machine learning models):

Continuous	Binary	Categorical
soil_nitrogen	*Antimalarial_Use*	*Genus (Target encoded)*
soil_ph	*Medicinal*	*Family (Target encoded)*
soil_ocs	*Wiki_Page*	*kg_mode (Target encoded)*
soil_water_cap	*Common_Name*	*Life-form (One hot encoded)*
soil_depth	*Tested_for_Alkaloids**	
bio1	*Poisonous*	
bio4	*Emergence*	
bio10	*In_Malarial_Region**	
bio11	*Activity_Antimalarial*	
bio12		
bio15		
bio16		
bio17		
elevation		
brkl_elevation		
slope		
latitude		
longitude		

2 TOOLS

To resolve names in data sources to accepted names in the World Checklist of Vascular Plants (WCVP) (Govaerts et al., 2021), we developed the `automatchnames` Python library[4]; and used `automatchnames v0.1` to resolve names to the WCVP V7. Methods used to collect and compile data are collected in the `miningtraitdata v0.1` repository.[5]

3 SOURCE DETAILS

3.1 Literature

Where we have relied on published literature for data, we have searched using Scopus, PubMed and Google Scholar. When searching for a particular property, we have searched this word along with every accepted genus in the three families of interest e.g. ‘antiplasmodial aspidosperma’. We have also searched for this word with each of the study family names.

[1] https://github.com/alrichardbollans/apocynaceae_rubiaceae_traits
[2] https://github.com/alrichardbollans/loganiaceae_traits
[3] https://github.com/alrichardbollans/antimalarial_trait_predictions
[4] https://github.com/alrichardbollans/automatchnames
[5] https://github.com/alrichardbollans/mining_trait_data

3.1.1 Ethnobotanical Data

References to medicinal usage on the Plants of the World Online (POWO, 2022) were collected using the `pykew` Python library[6] by searching for the terms: 'medicinal', 'medication' and 'medicine'. Similarly, references to antimalarial usage were collected using the terms: 'antimalarial' and 'malaria'.

The existence of Wikipedia[7] pages for species was determined by searching all species, subspecies and varieties (and their synonyms) using the `Wikipedia-API` Python wrapper.[8]

Common name data were compiled from the following sources (MPNS, 2022; USDA, 2022b; POWO, 2022; Kew Species Profiles, 2022; Plants and People Africa, 2022; Cornell College of Agriculture and Life Sciences, 2022; California Poison Control System, 2022; University of California Safe and Poisonous Garden Plants, 2022; USDA, 2022a; Günthardt et al., 2018; Wiersema and León, 2013) and Wikipedia[9]. Common name data from Wikipedia were extracted using the `wikipedia_searches` package in `miningtraitdata`. Data on common names were extracted from POWO by parsing the common name sections for each accepted species in Apocynaceae, Loganiaceae and Rubiaceae.

3.1.2 Phytochemistry

Metabolite data were extracted from KNApSAcK (Afendi et al., 2012) using the `metabolite_searches` package in `miningtraitdata` to search for all species, subspecies, varieties and their synonyms.

Data on poisonous plants were generated from (POWO, 2022; Diazgranados et al., 2020; Royal Botanic Gardens, Kew, 2021; Cornell College of Agriculture and Life Sciences, 2022; California Poison Control System, 2022; University of California Safe and Poisonous Garden Plants, 2022; USDA, 2022a; Günthardt et al., 2018; CliniPharm CliniTox, 2022) and Wikipedia. POWO references to poisonous plants were collected using the `pykew` library by searching for the terms 'poison', 'poisonous', 'toxic' and 'deadly'. Poisonous plants were extracted from the Wikipedia list of poisonous plants[10] as well as linked Wikipedia pages in other languages using the `Wikipedia-API`.

3.2 Morphology

References to emergences in POWO were collected using the `pykew` library by searching for the terms: 'hairs', 'hairy', 'pubescent', 'spine', 'thorn', 'spikes'.

The TRY database (Kattge et al., 2020) was used to collect data on the presence of emergences. Specifically, the data from hairs came from (Wirth and Lichstein, 2009; Wang et al., 2018; Prentice et al., 2011; Hoof et al., 2008; Hickler, 1999; Diaz et al., 2004; Blonder et al., 2016) and data on spines came from (Wang et al., 2018; van der Plas and Olff, 2014; Prentice et al., 2011; Onstein et al., 2014; Joseph et al., 2014; Frenette-Dussault et al., 2012; Diaz et al., 2004; Burrascano et al., 2015).

3.3 Geographic Regions with Malaria

Data on global malarial incidence and transmission were generated from (WHO, 2022; The World Bank, 2022; Centers for Disease Control and Prevention, 2022; Bryan et al., 1996; Girod et al., 1995; Lounibos and Conn, 2000; Snow et al., 2012; Chadee et al., 1993). The regions indicated in these sources have then been mapped onto the World geographical scheme for recording plant distributions (level 3) (Brummitt et al., 2001) and are indicated in Figure S1.

3.4 Occurrence Records

The GBIF Occurrence records used to generate the environmental data were gathered from the following (Gbif.Org (28 April 2022), 2022; Gbif.Org (29 April 2022), 2022; Gbif.Org (30 April 2022), 2022; Gbif.Org (01 June 2022), 2022c,b,a).

[6] `https://github.com/RBGKew/pykew`

[7] `https://www.wikipedia.org/` accessed on 14 Apr. 2022

[8] `https://github.com/martin-majlis/Wikipedia-API`

[9] `https://www.wikipedia.org/` accessed on 7 Oct. 2022

[10] `https://en.wikipedia.org/wiki/List_of_poisonous_plants` accessed on 8 Jun. 2022

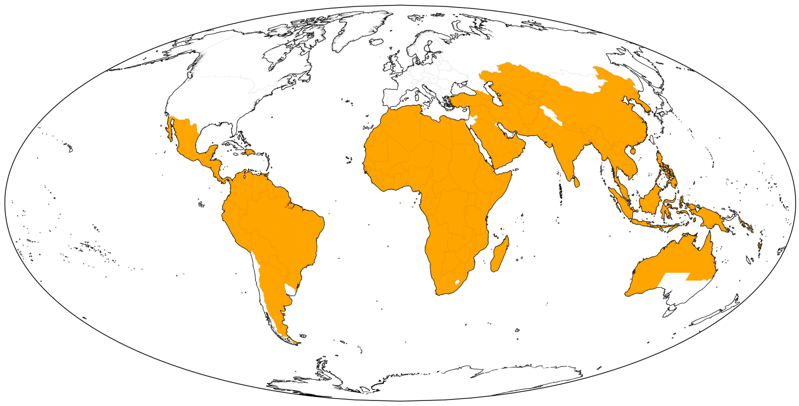

Figure S1. Malarial Regions (where transmission occurs).

3.5 Classifying Antiplasmodial Activity

Here we describe in detail the process for classifying plants as active or inactive from reported bioassays. Firstly, when conducting the literature review on antiplasmodial activity, we documented the authors' decisions regarding the degree of activity. Though authors use differing terminology and categories to label activity values, authors generally split into two or three categories — *Active*, *Weak* and *Inactive*. As we are using binary labels, *Active* and *Inactive*, in the following we consider *Weak* cases to be *Inactive*.

The main ambiguity that arose when parsing authors decisions was relating to the linguistic modifiers used. Any sort of neutral modifier (e.g. *Acceptable/Moderate* activity) was denoted as *Weak/Inactive*. Any positive modifier (e.g. *Strong*, *Good* etc..) was denoted as *Active*. Next we documented the given values for activity and the type of test used, including the strain of malaria, plant part and preparation method.

To provide a binary classification of the given activity values we separated into three main cases, Crude Extractions, Fractions and Isolations, which are further subdivided into *in vitro* and *in vivo* tests. These are further divided based on measurement units.

Crude Extractions

In *in vitro* cases where authors provide definite IC50 values in $\mu g/ml$, we follow a generalised version of the definitions given in (Rasoanaivo et al., 2004).[11] Extracts with activities of $< 10\mu g/ml$ are considered to warrant further investigation, so we label samples as follows:

- $< 10\mu g/ml$ *Active*
- $> 10\mu g/ml$ *Inactive*

This corresponds well to most authors interpretations.

Sometimes authors provide degrees of inhibition at differing doses rather than IC50 values. In clear active cases (e.g. 80% inhibition at 9 $\mu g/ml$) and inactive cases (22.35% inhibition at 100 $\mu g/ml$), we use the above schema. When there is ambiguity we use authors decisions.

In *in vivo* contexts, there is not a standardised dosing across studies, and cytotoxicity often influences authors' judgements. Rasoanaivo et al. (2004) provide suggested classifications for inhibition rates at $250mg/kg/day$ but it is not clear how to translate these to studies in the literature. Authors decisions are used here.

Fractions

Tests using fractions were relatively rare. For a plant containing an active compound, one would expect a fractionation containing this compound to be more active than the crude extracts. To reflect this, we use an IC50 threshold of 5 $\mu g/ml$. This threshold in general corresponds to authors decisions.

[11] 'Very Good':< 0.1, 'Good':$0.1 - 1.0$, 'Moderate':$1.1 - 10$, 'Weak':$11 - 25$, 'Very Weak':$26 - 50$, 'Inactive': > 100

Isolated Compounds

According to the the Medicines for Malaria Venture[12] compounds with IC50 values under 1 μM are of interest for further investigation. Where compounds have been isolated from plants and subsequently tested, we use this threshold in our data for *in vitro* studies.

Known Antiplasmodial Compounds

Where isolated compounds have been found to be active, we have cross-referenced these compounds with the presence of compounds in other species using KNApSAcK (Afendi et al., 2012) and labelled those species containing these compounds as active. All such species had in fact also been tested for their antiplamsodial activity in bioassays and so the inclusion of data on antiplasmodial compounds has not affected knowledge of the sampling biases.

Final Decision

For each plant we obtain a list of tests for antiplasmodial activity and their associated activity labels. In cases of plants with multiple tests, we assign the label *Active* if any of the tests are active.

REFERENCES

Afendi, F. M., Okada, T., Yamazaki, M., Hirai-Morita, A., Nakamura, Y., Nakamura, K., et al. (2012). KNApSAcK family databases: integrated metabolite–plant species databases for multifaceted plant research. *Plant and Cell Physiology* 53, e1–e1. Publisher: Oxford University Press

Blonder, B., Baldwin, B. G., Enquist, B. J., and Robichaux, R. H. (2016). Variation and macroevolution in leaf functional traits in the Hawaiian silversword alliance (Asteraceae). *Journal of Ecology* 104, 219–228. doi:10.1111/1365-2745.12497

Brummitt, R. K., Pando, F., Hollis, S., and Brummitt, N. (2001). *World geographical scheme for recording plant distributions*, vol. 951 (International working group on taxonomic databases for plant sciences (TDWG))

Bryan, J. H., Foley, D. H., and Sutherst, R. W. (1996). Malaria transmission and climate change in Australia. *Medical Journal of Australia* 164, 345–347. doi:10.5694/j.1326-5377.1996.tb122051.x

Burrascano, S., Copiz, R., Del Vico, E., Fagiani, S., Giarrizzo, E., Mei, M., et al. (2015). Wild boar rooting intensity determines shifts in understorey composition and functional traits. *Community Ecology* 16, 244–253. doi:10.1556/168.2015.16.2.12

[Dataset] California Poison Control System (2022). `https://calpoison.org/topics/plant`. Accessed: 2022-02-22

[Dataset] Centers for Disease Control and Prevention (2022). Where Malaria Occurs. `https://www.cdc.gov/malaria/about/distribution.html`. Accessed: 2022-05-12

Chadee, D. D., LeMaitre, A., and Tilluckdharry, C. C. (1993). An epidemic outbreak of plasmodium vivax malaria in Trinidad-abstract. *West Indian med. j* , 45–6

[Dataset] CliniPharm CliniTox (2022). `https://www.vetpharm.uzh.ch/perldocs/toxsyqry.htm`. Accessed: 2022-02-23

[Dataset] Cornell College of Agriculture and Life Sciences (2022). `http://poisonousplants.ansci.cornell.edu`. Accessed: 2022-02-22

Diaz, S., Hodgson, J., Thompson, K., Cabido, M., Cornelissen, J., Jalili, A., et al. (2004). The plant traits that drive ecosystems: Evidence from three continents. *Journal of Vegetation Science* 15, 295–304. doi:10.1111/j.1654-1103.2004.tb02266.x

Diazgranados, M., Allkin, B., Black, N., Cámara-Leret, R., Canteiro, C., Carretero, J., et al. (2020). World checklist of useful plant species Publisher: KNB Data Repository

Frenette-Dussault, C., Shipley, B., Léger, J.-F., Meziane, D., and Hingrat, Y. (2012). Functional structure of an arid steppe plant community reveals similarities with Grime's C-S-R theory. *Journal of Vegetation Science* 23, 208–222. doi:10.1111/j.1654-1103.2011.01350.x

Gbif.Org (01 June 2022) (2022a). Occurrence Download (Loganiaceae Species) doi:10.15468/DL.SJ97KJ

Gbif.Org (01 June 2022) (2022b). Occurrence Download (Loganiaceae Subspecies) doi:10.15468/DL.8EJG4Q

[12] `https://www.mmv.org/20th-call-proposals` accessed on 30 Aug. 2022.

Gbif.Org (01 June 2022) (2022c). Occurrence Download (Loganiaceae Varieties) doi:10.15468/DL.PMGJCD

Gbif.Org (28 April 2022) (2022). Occurrence Download (Apocynaceae and Rubiaceae Species) doi:10.15468/DL.MXEE8H

Gbif.Org (29 April 2022) (2022). Occurrence Download (Apocynaceae and Rubiaceae Subspecies) doi:10.15468/DL.X4GTKM

Gbif.Org (30 April 2022) (2022). Occurrence Download (Apocynaceae and Rubiaceae Varieties) doi:10.15468/DL.3EUP4A

Girod, R., Salvan, M., and Denys, J. (1995). Control of malaria re-emergence in Reunion. *Sante (Montrouge, France)* 5, 397–401

Govaerts, R., Nic Lughadha, E., Black, N., Turner, R., and Paton, A. (2021). The World Checklist of Vascular Plants, a continuously updated resource for exploring global plant diversity. *Scientific Data* 8, 1–10. doi:https://doi.org/10.1038/s41597-021-00997-6. Publisher: Nature Publishing Group

Günthardt, B. F., Hollender, J., Hungerbühler, K., Scheringer, M., and Bucheli, T. D. (2018). Comprehensive Toxic Plants–Phytotoxins Database and Its Application in Assessing Aquatic Micropollution Potential. *Journal of Agricultural and Food Chemistry* 66, 7577–7588. doi:10.1021/acs.jafc.8b01639

Hickler, T. (1999). *Plant functional types and community characteristics along environmental gradients on Öland's Great Alvar (Sweden)*. PhD Thesis, Master's thesis, University of Lund, Sweden

Hoof, J., Sack, L., Webb, D. T., and Nilsen, E. T. (2008). Contrasting Structure and Function of Pubescent and Glabrous Varieties of Hawaiian Metrosideros polymorpha (Myrtaceae) at High Elevation. *Biotropica* doi:10.1111/j.1744-7429.2007.00325.x

Joseph, G. S., Seymour, C. L., Cumming, G. S., Cumming, D. H., and Mahlangu, Z. (2014). Termite Mounds Increase Functional Diversity of Woody Plants in African Savannas. *Ecosystems* 17, 808–819. doi:10.1007/s10021-014-9761-9

Kattge, J., Bönisch, G., Díaz, S., Lavorel, S., Prentice, I. C., Leadley, P., et al. (2020). TRY plant trait database–enhanced coverage and open access. *Global change biology* 26, 119–188. Publisher: Wiley Online Library

[Dataset] Kew Species Profiles (2022). Species Profile Vernacular. Accessed: 2022-01-18

Lounibos, L. P. and Conn, J. E. (2000). Malaria Vector Heterogeneity in South America. *American Entomologist* 46, 238–249. doi:10.1093/ae/46.4.238

[Dataset] MPNS (2022). Medicinal Plant Names Services, Version 11. Publisher: Royal Botanic Gardens, Kew, accessed on 18/01/2022

Onstein, R. E., Carter, R. J., Xing, Y., and Linder, H. P. (2014). Diversification rate shifts in the Cape Floristic Region: The right traits in the right place at the right time. *Perspectives in Plant Ecology, Evolution and Systematics* 16, 331–340. doi:10.1016/j.ppees.2014.08.002

[Dataset] Plants and People Africa (2022). Common Names from Plants and People Africa. `http://www.plantsandpeopleafrica.com/`. Accessed: 2022-01-10

[Dataset] POWO (2022). Plants of the World Online. Facilitated by the Royal Botanic Gardens, Kew. `http://www.plantsoftheworldonline.org/`

Prentice, I. C., Meng, T., Wang, H., Harrison, S. P., Ni, J., and Wang, G. (2011). Evidence of a universal scaling relationship for leaf CO_2 drawdown along an aridity gradient. *New Phytologist* 190, 169–180. doi:10.1111/j.1469-8137.2010.03579.x

Rasoanaivo, P., Deharo, E., Ratsimamanga-Urveg, S., and Frappier, F. (2004). Guidelines for the nonclinical evaluation of the efficacy of traditional antimalarials. In *Traditional Medicinal Plants and Malaria* (CRC Press). 324–341

Royal Botanic Gardens, Kew (ed.) (2021). *LitTox database* (London: Royal Botanic Gardens, Kew)

Snow, R. W., Amratia, P., Kabaria, C. W., Noor, A. M., and Marsh, K. (2012). The Changing Limits and Incidence of Malaria in Africa. In *Advances in Parasitology* (Elsevier), vol. 78. 169–262. doi:10.1016/B978-0-12-394303-3.00010-4

[Dataset] The World Bank (2022). World Development Indicators. `https://datacatalog.worldbank.org/search/dataset/0037712`. Accessed: 2022-05-03

[Dataset] University of California Safe and Poisonous Garden Plants (2022). `https://ucanr.edu/sites/poisonous_safe_plants/`. Accessed: 2022-02-22

[Dataset] USDA (2022a). Dr. Duke's Phytochemical and Ethnobotanical Databases. `http://dx.doi.org/10.15482/USDA.ADC/1239279`. Accessed: 2022-02-22

[Dataset] USDA (2022b). The PLANTS Database. http://plants.usda.gov. Accessed: 2022-01-10

van der Plas, F. and Olff, H. (2014). Mesoherbivores affect grasshopper communities in a megaherbivore-dominated South African savannah. *Oecologia* 175, 639–649. doi:10.1007/s00442-014-2920-z

Wang, H., Harrison, S. P., Prentice, I. C., Yang, Y., Bai, F., Togashi, H. F., et al. (2018). The China Plant Trait Database: toward a comprehensive regional compilation of functional traits for land plants. *Ecology* 99. doi:10.1002/ecy.2091

[Dataset] WHO (2022). Global health observatory: Number of indigenous malaria cases. https://www.who.int/data/gho/data/indicators/indicator-details/GHO/number-of-indigenous-malaria-cases. Accessed: 2022-09-14 (World Health Organization)

Wiersema, J. and León, B. (2013). *World Economic Plants: a standard reference* (CRC Press)

Wirth, C. and Lichstein, J. W. (2009). The Imprint of Species Turnover on Old-Growth Forest Carbon Balances - Insights From a Trait-Based Model of Forest Dynamics. In *Old-Growth Forests*, eds. C. Wirth, G. Gleixner, and M. Heimann (Berlin, Heidelberg: Springer Berlin Heidelberg), vol. 207. 81–113. doi:10.1007/978-3-540-92706-8_5. Series Title: Ecological Studies
